# State strategies of governance in biomedical innovation: aligning conceptual approaches for understanding 'Rising Powers' in the global context

**DOI:** 10.1186/1744-8603-7-3

**Published:** 2011-02-24

**Authors:** Brian Salter, Alex Faulkner

**Affiliations:** 1 Department of Political Economy, King's College London, Strand, London WC2R 2LS, UK

## Abstract

**Background:**

'Innovation' has become a policy focus in its own right in many states as they compete to position themselves in the emerging knowledge economies. Innovation in biomedicine is a global enterprise in which 'Rising Power' states figure prominently, and which undoubtedly will re-shape health systems and health economies globally. Scientific and technological innovation processes and policies raise difficult issues in the domains of science/technology, civil society, and the economic and healthcare marketplace. The production of knowledge in these fields is complex, uncertain, inter-disciplinary and inter-institutional, and subject to a continuing political struggle for advantage. As part of this struggle, a wide variety of issues - regulation, intellectual property, ethics, scientific boundaries, healthcare market formation - are raised and policy agendas negotiated.

**Methods:**

A range of social science disciplines and approaches have conceptualised such innovation processes. Against a background of concepts such as the competition state and the developmental state, and national innovation systems, we give an overview of a range of approaches that have potential for advancing understanding of governance of global life science and biomedical innovation, with special reference to the 'Rising Powers', in order to examine convergences and divergences between them. Conceptual approaches that we focus on include those drawn from political science/political economy, sociology of technology; Innovation Studies and Science & Technology Studies. The paper is part of a project supported by the UK ESRC's Rising Powers programme.

**Results:**

We show convergences and complementarities between the approaches discussed, and argue that the role of the national state itself has become relatively neglected in much of the relevant theorising.

**Conclusions:**

We conclude that an approach is required that enables innovation and governance to be seen as 'co-producing' each other in a multi-level, global ecology of innovation, taking account of the particular, differing characteristics of different emerging scientific fields and technologies. We suggest key points to take account of in order in the future to move toward a satisfactory integrative conceptual framework, capable of better understanding the processes of the emergence, state steerage and transnational governance of innovative biomedical sectors in the Rising Powers and global context.

## Introduction

Biomedical innovation has become an accepted priority in the industry policies of most of the advanced, developed societies. The emergence of a wide range of innovative bio-industries, including the cell and tissue therapies of regenerative medicine, molecular diagnostics and genetic tests, biopharmaceuticals and medical devices, takes place under conditions of globalisation. The geopolitics of how biomedical innovation is and should be governed at state, regional and transnational levels is now an embedded feature of the bioeconomy. With the rapid rise of, especially, China and India as global players in the life sciences, the long-established hegemony of the United States (US) in this field [1] is being challenged, at least in terms of political intent, with a consequent intensification of the struggle over what forms of governance should be introduced by whom, where, when and how. As a result, a new political dynamic is being created as states, multi-national corporations and civil society organisations compete for position in the existing and emerging governance space of the biomedical knowledge economy.

In this paper, we provide an overview of several strands of scholarship that bear on these issues in order to elicit the primary dimensions which are necessary in the future to go on to build a conceptual framework to guide and inform debate about how best to analyse this phenomenon. The aim is thus the relatively modest one of identifying, describing, mapping and aligning a set of relevant approaches, as a prelude to what would be a further, new project in conceptual framework-building. The focus is on the nexus between innovation and governance at national, transnational and global levels, giving examples from the field of biomedicine. The overview and discussion is organised under the topics listed below. The first four sections provide a substantive political science-based mapping of state-of-the-art analytic approaches to understanding of the state politics of innovation and governance applying to global biomedicine, and to the emerging global political dynamic associated with the so-called Rising Powers (examples being given broadly but with an emphasis on India and China). This is followed by two sections providing an overview of relevant concepts and approaches drawn from innovation studies and Science & Technology Studies, which provide a complementary set of concepts for investigating innovation-governance processes. Attention is drawn to points at which these different sets of concepts converge and overlap.

• States, politics and innovation

• States, politics, and multidimensional governance of biomedical innovation

• States, politics and the transnational governance of biomedical innovation

• The Rising Powers and hegemonic challenge in biomedical innovation

• Innovation ecologies and pathways

• Co-production of innovation-governance; technological zones as emerging sectors

• Conclusions: the conceptual challenge for global biomedical innovation-governance

## States, politics and innovation

In an analysis of the political economy of the development of medicine, the competition for control of biomedical innovation governance is driven by the anticipated demand of future populations for improved and more efficient healthcare, the future knowledge market generated by this demand and the economic benefits that will accrue to those able to shape access to that market to their advantage. In this context, the approach adopted by states to innovation governance forms an integral part of political ambition.

In general terms, the advanced economies of North America and Europe met the uncertainties accompanying the shift from Fordist to post-Fordist modes of mass production and consumption with evolution of the 'competition' state as the vehicle for the pursuit of national advantage through innovation [2-4]. Rather than concerning themselves with government interventions to ensure full employment and respond to market failures, states began to focus their attention instead on the neo-liberal supply-side policies that would give a sharp edge to their competitiveness in the global knowledge economy. Particularly in the case of the knowledge driven bio-industries, this meant a concentration not only on the infrastructures of innovation but also on 'agglomeration and network economies and the mobilisation of social as well as economic sources of flexibility and entrepreneurialism' [5]. To be effective as players in the global economy, it was argued, competition states needed to bring their social and cultural values into line with their entrepreneurial ambitions. Entrepreneurialism needed to be embedded: as a consequence the institutional reforms of the competition state eschewed rigid bureaucratic hierarchies and relied instead on new forms of network based governance [6,7].

In deciding how to intervene and with what policies, this form of analysis has shown that competition states of the West have moved away from the national sponsorship of particular firms and technologies and towards policies designed to foster 'the conditions necessary for innovation', although this model is challenged by the recent credit crisis impacting on infrastructure investments. In common with innovation theorists, they recognise that the concept of a self-contained national innovation system is no longer tenable either empirically or as a policy objective. Hence, the goal has become less one of specific structural change and more one of stimulating a dynamic that enables the knowledge production process to become self-sustaining. It has become part of the established policy orthodoxy that regional (sub-national or trans-national) governments should initiate programmes to foster cluster developments in sectors such as biotechnology [8]; that commercialisation can be facilitated through academic-industry collaborations and high profile, publicly funded R & D centres acting as magnets for venture capital investment [9-11]; that networks of science and industry should be enabled; that regulation should be facilitative rather than restrictive [12]; and that intellectual property rights (IPR) should favour the operation of the market. In this conceptual approach, as with all orthodoxies, there is the expectation of policy convergence between states characterised by assumptions regarding 'best practice' in the field [13].

Not all governments adopted this competition state model in response to the challenges of globalisation. Shaped by a different historical experience and lacking the scientific critical mass and innovation infrastructures of competition states, the states of the developing world chose a different approach. Focusing in the main on South Korea, Taiwan, Japan, and to some extent Singapore, in the 1980s and early 1990s, the earlier work on the 'developmental state' highlights its role in the promotion of rapid economic development through the targeting of particular industries with large global markets. The markets were already there. The political task was to penetrate them. To achieve this goal, the state protected its chosen industries using a range of policies such as import and credit controls, promoted them through state investment, guided private capital through incentive schemes, and measured their progress in terms of export achievements [14-16]. Backed by a strong, professional and autonomous bureaucracy, the state sought to define the specific path of industrialisation through the 'government of the market' [17,18].

In this analysis, the essence of those states' commonality is that they sought to challenge the control exercised by the developed world over the dynamic of globalisation. If they were to access the wealth of global markets, if they were to 'catch up' with Western countries, then the power of the state was required to make globalisation work for them. However, having caught up using the targeting of known markets as a primary policy objective, developmental states face the problem of 'keeping up' in the context of future markets like the life sciences that are either unknown or decidedly uncertain [19]. Like competition states, they are conceived to be obliged to adapt their strategies in the light of both threats and opportunities in the international economy [20]. This approach highlights that they are also aware that, in the case of biomedicine and the life sciences in general, that their traditional modes of direct state intervention do not suit the innovation requirements of what is an elusive science with a speculative future, an uncertain market and a difficult path to commercialisation. As we shall see in later sections, nascent and emerging technological sectors and zones tend to manifest their own particular characteristics and governance requirements. As a consequence, observers taking a political economy perspective have noted the evolution of developmental state governance into new forms described variously as the 'adaptive state', the 'flexible state', the 'post-industrial developmental state', the 'transformative state' and the 'catalytic state' in their studies of Japan, South Korea and Taiwan [21-23]. As Wong observes of biotechnology in Taiwan, it is a case of the state identifying 'the right mix of public policies aimed at facilitating technology innovation and knowledge-based interventionist strategies' and recognising that 'cutting edge technologies can no longer be borrowed; rather they must be created' - which means a change in state direction and an investment in, or access to, basic science [[24]: 169-70]. In moving from borrowers to innovators, this view focuses attention on how developmental states are obliged to review the extent of their autonomy and the style of the bureaucracy that implements their policies.

## States, politics and multidimensional governance of biomedical innovation

The implications of the complexities of innovation for the role of the state in the governance of biomedical knowledge production are considerable, as we explore in more detail later in the paper, drawing on innovation studies and STS theory. States have a wide range of policy choices regarding the point and mode of intervention in this process in terms of the science itself, society and the future market. Precisely what policies should be introduced to support a state's ambition is not clear and will be driven in part by how they seek to position themselves within the global value chain of innovation and in part by the level of development of the sector or zone. For novel fields of biomedicine, the knowledge production process from the basic science, through clinical experimentation and trials, to the therapeutic product is long, arduous and uncertain. At all stages in that process, there exists a potential triangle of tensions that can be summarised as being between science, society and the market [25]: the science may prove to be inadequate, society unsympathetic or the market uninterested. As a result there is pressure for governance to be 'co-produced' with science in response to these tensions. As Jasanoff puts it, this means a focus on how 'knowledge making is incorporated into practices of state-making, or of governance more broadly, and in reverse, how practices of governance influence the making and use of knowledge' [26]. Thus, the chosen mode of governance can be critical in determining the success or failure of the biomedical innovation. This key dynamic and the conceptual approach that represents it is discussed in more detail below, in the sections on Innovation ecologies and Co-production.

If innovation policy is to be successful, this triangle of tensions highlights the following crucial dynamics. First, choices about the *science *have much to do with the creation and husbanding of the resources necessary for the enterprise to have an explicit domestic platform. This may require investment, an adequate research funding market and an appropriate supply of scientific labour and research materials such as oocytes and stem cell lines. Secondly, regardless of political system, the response of *society *to biomedical science, for example as mobilised through civil society organisations or in the formation of ethical positions, may require choices to be made about how that response is negotiated both domestically and internationally if public trust in the field is to be maintained. As we discuss below, public expectations and imagined futures play a potent role in the progress a given area of science or emerging sector may or may not make. Even if, as in China, the public voice is muted, both elite and international opinion nonetheless act to request, if not require, policies that regulate the science in the public interest - not only in terms of risk and safety but also the sensitivities of cultural values. Without such policies, future consumer demand may be fatally undermined. Thirdly and finally, the risk of *market *failure during the long gestation from basic science to eventual therapy means that early government funding intervention may be necessary to motivate patenting, venture capital investment and pharmaceutical engagement in this emerging industry. To simplify a complex set of institutional positions, for countries such as the UK, it is primarily the state which is seen as the appropriate agent for interventions in the innovation process with private interests playing a relatively lesser role, whereas in the case of the US, this equation may be reversed in many, though not all, biomedical sectors.

At the same time, the perspective demonstrated in the above discussion strongly indicates that if the construction of global advantage by a state is the goal, it must recognise that: (a) the national-regional levels of governance play different and potentially complementary roles; (b) direct public intervention may be counterproductive and at the very least needs to be matched by indirect policies aimed at stimulating private sector involvement; and (c) private governance e.g. (organised self-regulation by producers via voluntary codes of practice and standards) is a necessary part of the policy package. What is being described here is of course a form of multi-level governance where the dispersion of governance across several jurisdictions has the assumed advantage that it is more flexible than the concentration of governance in the one jurisdiction of the central state [[27]: 235]. Against this advantage has to be set the possible transaction costs of coordinating multiple jurisdictions, should this prove necessary. In addition, not all of these jurisdictions may be responsive to state suggestions. The expansion of private governance as a result of the complexities of scientific advance and the need for continuous technical and ethical rule making has established a realm of governance with its own networks, authorities, and procedures [28-31].

The combination of the 'triangle of tensions' with multiple levels of governance leads to an initial framework for a structural and process analysis of innovation governance, which is outlined in our Conclusion, below. Having focused in this section on the broad *dimensions *of a governance analysis, the following section now focuses upon the *transnational nature *of the governance space that competition states and the Rising Powers must build and inhabit.

## States, politics and the transnational governance of biomedical innovation

The competition between states for advantage in the transnational markets that constitute the global infrastructure of biomedical knowledge production (research funding, scientific labour, moral economy, intellectual property and VC finance) takes place within a complex and very fluid transnational governance and regulatory space that has the capacity to direct, steer or ignore the operation of these innovation markets. It is in the political interest of states to seek to shape that transnational governance space in ways that enhance the synergistic engagement of the markets with their own national innovation strategies.

As we explore in detail in the next section, the political economy perspective highlights how national states will bring quite different historical perspectives to bear on the question of where and how to intervene in the transnational governance space of biomedical innovation. For states of the developed world it is likely to be in their best interest to support governance measures that reinforce the model of innovation that has served them well in the past. For the developing economies, on the other hand, approaches to innovation based on their particular strengths and weaknesses will lead them to influence transnational governance in a different direction. Commenting on the 'sclerotic' qualities of the established drug innovation model traditionally sponsored by the US and European Union, Tait observes of China and India that 'these increasingly powerful components of the bioeconomy may see a competitive advantage in leading regulatory reform so as to encourage more innovative health care sectors to develop, initially for their large and increasingly wealthy home markets, and perhaps also to encourage change in the United States and European regulatory systems' [32]. Given their global ambition, China and India must also target the apparatus of transnational governance.

In so doing, they are faced with a wide range of possible points of intervention in the governance space, which includes formal international institutions, intergovernmental agreements, self-regulatory or private regimes, combined public/private networks, and what Coleman terms 'loose couplings' of allied interests on particular issues [33]. In some cases the apparatus of governance is highly developed, institutionalized, and underpinned and legitimised by international legal agreement. In others it consists of a transnational network that contributes to the process of 'governance without government' through non-hierarchical forms of steering by private actors and the creation of non-state forms of political order [34-37]. As Rosenau describes the contribution of such a network, 'Here governance is conceived as systems of rule, as the purposive activities of any collectivity that sustain mechanisms designed to insure its safety, prosperity, coherence, stability and continuance' [[38]: 171]. It is what he terms a 'horizontal network' with authority embedded in the informal rules through which it conducts its affairs [[38]: 191]. Such is the variety of the types of governance that can inhabit and interact in the transnational space of global activities that a tendency is produced toward governance that is both multidirectional and a mixture of formal and informal structures 'in which the dynamics of governance are so intricate and overlapping among the several levels as to form a singular, web-like process that... neither begins nor culminates at any level or at any point of time' [39]. This form of governance coincides with the transnational character of technology innovation which, as is discussed below, readily flows across national jurisdictions. This principle is also shown in Science & Technology Studies approaches to standardisation and 'portability' of innovative scientific knowledge, a point of conceptual convergence in the approaches that we illustrate in this paper. This is discussed further in the sections below dealing with innovation ecologies and the concept of the co-production of innovation and governance.

For those engaged in the competition for future position in biomedical innovation, these many points of possible intervention are not neutral arenas of dispassionate governance debate but political sites where alliances are formed in order to protect and further particular interests. For example, the development of a transnational form of patenting governance through the Agreement on Trade Related Intellectual Property Rights (TRIPS) has been characterised by a continuing conflict between the interests of developed and developing world states. It is inevitable that states at different stages of economic development and consequently with their own particular approaches to the exploitation of the innovation value chain will place different political demands on global patenting governance. As a form of transnational governance in biomedical innovation, TRIPS is characterized by its international legal status as a vehicle for multi-level governance to standardize the operation of the market, the legitimizing power of the WTO, bureaucratic complexity, and continuing and highly visible political conflict. A contrasting example of transnational governance is provided by the work of the International Stem Cell Forum, established by the UK Stem Cell Bank (UKSCB) as a vehicle for standardization in stem cell science [40]. Here we have a private, non-government, mode of network governance, motivated by the desire of science for a form of self-regulation that will enhance its capacity to operate transnationally. As noted in the previous paragraph, the important issue of standardization as a mode of non-governmental governance is one of the points of alignment between political science approaches and those from Innovation Studies and Science and Technology Studies (STS).

## The Rising Powers and hegemonic challenge in biomedical innovation

This section now turns to consider the political economy perspective as applied specifically to the substantive issue of the US position in the global bioeconomy, and the Rising Powers' strategic response to this. The drive by some developing countries to establish a governance platform for a competitive entry into the global biomedical economy collides with the reality of US hegemony in the model and practice of biomedical innovation. America's powerful mix of federal, state and private funding for biomedical science resonates easily with its dominant model of how the governance of innovation in the biotechnology industry facilitates the movement from basic science to commercial product: a model that the US has propagated energetically on the global stage [25]. It assumes a strong and established infrastructure of scientific research supported by a clear market emphasis on the facilitation of a close engagement between academy and industry in order to provide ideological and practical support for research applications [41,42]. The US state created a permissive patenting regime with the capacity to support and enhance its ambitions for a global biomedical industry [43]. As a result, university technology transfer offices in the US focus on helping their scientists to patent their findings, identify private investment opportunities and negotiate advantageous intellectual property agreements with their scientific partners in developing countries [44]. Such is the power of the US model of biotech innovation that it enables the US easily to sustain its position when measured on all the primary indicators of innovation capacity in this field: R and D investment, concentration of research, scientific workforce, experimental trials by SMEs, supporting IP laws, venture capital investment and potential healthcare market [1,25].

In seeking to challenge the US hegemony, developmental states have had to adapt their modus operandi to the needs of an uncertain knowledge market [45,46] and to move towards what have been described as 'ecologies of innovation' (see below). The precise mode of adaptation varies and is dependent on local political conditions, a realistic assessment of their innovation strengths and weaknesses, the regional context, and decisions regarding the strategic entry points on the biotechnology value chain of innovation that a state deems appropriate [47]. In Singapore, the state retains tight control in its pursuit of economic prosperity through the promotion of the biomedical industry. It remains a single party state with a high level of state ownership that still prohibits public demonstrations, exercises a tight labour control scheme, and limits public debate [48]. Yet at the same time its bureaucracy has adapted itself in order to 'enable' the emergence of its health technology economy by providing research facilities, funding, and a supportive regulatory climate responsive to international requirements [49]. It may have moved towards a more liberal economy, but on the state's terms. Contrast this with the situation in Taiwan where, in the promotion of biotechnology, the earlier cohesive bureaucracy has been replaced by a proliferation of government agencies characterised by overlapping and competing jurisdictions [24]. Or again, South Korea where the growth of working class and bourgeoisie opposition combined with the divisions between the *chaebol *(family controlled corporate groups) and the state to reduce the traditional autonomy of the latter [50,51]. Nonetheless, despite their considerable differences, all three developmental states have taken the view that their national innovation strategies on health biotechnology should form part of a larger, global enterprise. With the effects of the WTO's initiatives on international economic governance feeding through the global system, developmental states are obliged to be proactive in their approach to globalisation regardless of their earlier leanings towards protectionism.

The Rising Powers of China and India, as relatively recent emerging economies, bring a different perspective to the developmental state approach as a means for achieving geopolitical advantage. Unlike Singapore, South Korea, Taiwan and Japan, they did not experience the rapid economic growth of the 1980s and early 1990s. For China, the massive shift from a centrally planned to a market-influence economy that commenced in 1978 required a seismic change in the role of the state that only gathered real momentum in the mid-1990s. Membership of the WTO and exposure to the pressures of global competition in the early 1990s caused what Saich has described as 'the internationalisation of the reform process' and an intensification of demands for the state to move from a directive to a facilitative role [[52]:4]. In practice, although the level of administrative intervention has diminished, the state in China for example has retained many of its old monopoly functions whilst simultaneously expanding its role into the new regulatory policy arenas needed to participate effectively in the global knowledge economy [52-54]. Its infrastructure needs as a player in this economy remain considerable and it is still in important respects playing 'catch up' [55]. India, meanwhile, is a revealing example of a country that initially took the traditional developmental state path but, by the early 1990s, had clearly failed to meet its economic objectives [56,57]. With impressive adaptability, the Indian state subsequently reconstructed itself as the enabling vehicle for a deregulated economy that has produced the explosive growth of the last 15 years [58,59].

The degree of adaptability of developmental states is a critical political factor in their response to the economic possibilities of biomedical innovation [60] because it must acknowledge the fluidity of that phenomenon be it expressed through technological sectors, zones or networks (see section below, Co-production). Lacking the resources to compete for global advantage across the full range of policy components that shape the knowledge production process in this field, developmental states must necessarily be selective, choosing their points of policy intervention in the light of their strengths and weaknesses and consequently how they seek to position themselves in the global game. As the World Bank report *China and the knowledge economy: seizing the 21*^*st *^*century *observes, 'developing countries do not have to reinvent the wheel: there are many ways for them to tap into and use the knowledge created in developed countries' [55]. In this respect a state's territorial jurisdiction of knowledge is less important than its strategic capacity for global access to the components of knowledge production.

## Innovation ecologies and pathways

In this section, having reviewed and illustrated state-of-the-art political economy understanding of the competitive nation-state politics of biomedical innovation under globalisation, we turn to a review of concepts from innovation studies and Science & Technology Studies (STS), to see how they can link with and contribute to developing an understanding of both state and non-state dynamics in shaping innovation and governance trajectories in the biomedical field. As we show, there are a number of key convergences between the political economy-based conceptual analysis illustrated above and those now to be discussed. These convergences can be seen most obviously in the analysis of the 'modular' model of innovation. Various concepts have been and are being used to understand scientific, industrial, and technological innovation processes, in a vast and diverse set of literatures in innovation studies and STS. These concepts tend to be generic, in the sense that they are not derived from observation of trends in the *realpolitik *of inter-national innovation/governance and states' relationships in the way that the previous sections of this paper have discussed. Many of the approaches owe some of their origins to evolutionary economics, and many of them have a tendency to position the governance work of the state as one of a variety of 'institutional' actors which are typically conceived of as lying outside innovation processes themselves. However, these 'institutional' factors have now been recognised as requiring further research attention amongst innovation studies scholars.

Relevant concepts from innovation studies include: sectoral innovation systems; technological systems; sociotechnical systems; technological regimes; and technological paradigms. The long-established concept of 'national innovation system' [61] does focus on national processes, having been used to show, for example, how the nature and degree of national state coordination of the development of biotechnology varied between the UK, US and Japan [62]. The sectoral systems approach in contrast 'places emphasis on the role of non-firm organizations such as universities, financial institutions, government, local authorities and of institutions and rules of the games such as standards, regulations, labor markets and so on'. As Malerba [63] notes, these differ greatly in their configuration across sectors, and affect the 'innovative, and productive activities of firms'. Noting that the understanding of 'institutions' effect on sectoral innovation is in its infancy, Malerba [64] has outlined future directions for research that should highlight public policy interventions which shape the growth and transformation of sectoral systems processes. The concept of sector obviously extends beyond nation-state boundaries, and variation in 'rules of the game' between different sectors has important implications for states and others' governance strategies. The articulation between state political strategies and sectoral dynamics is thus a key point of convergence between the different approaches discussed here, where further systematic conceptual development remains to be undertaken.

The definition of 'technological system,' has been stated to be a 'network of agents interacting in specific economic/industrial area under a particular institutional infrastructure...' [65]. On the other hand, the concept of 'technological regimes' [66-69] has been developed as being more explicitly technology-oriented and more concerned with knowledge and learning, taking as its focus the possibilities that a technology's actors envision as feasible in the development of a technology, and highlighting the ease with which knowledge can be shared - partly dependent on the extent to which it is tacit or codified [69]. This approach distinguishes especially between 'science-based' and 'cumulative' technologies and highlights the perspective of the entrepreneurial firm. This distinction is of clear importance in examining different strands of biomedical innovation, for example in contrasting the biotech (science) and medical device (cumulative) sectors. Thus here we have conceptual approaches that seek to understand the dynamics of discrete technologies as packages of knowledge, expertise and labour associated with their own individual dynamics. These concepts can be positioned as complementary to the foregoing political economy analysis of governance strategies for supporting the conditions of innovation, by providing a focus on partly historical characteristics of technologies and emergent sectors themselves. Such characteristics must be understood by any nation state seeking competitive or collaborative advantage through enterprise and regulatory policymaking.

In contrast to these systems-oriented concepts, 'ecology' has been used as a metaphor in a variety of fields related to scientific innovation and medicine in order to capture the complexity, contingency, and indeterminacy associated with the process of knowledge production or technology development and diffusion [70,71]. Thus, important work in innovation systems analysis applied to medical technology innovation has conceptualised innovation in this way [72], moving towards a more plural, polycentric and interactive view of medical scientific innovation, as well as demonstrating empirically the distributed nature of biomedical innovation networks as they shift and evolve over time and place, involving scientific and practical knowledge that cuts across established boundaries of expertise and nation-state [73,74]. These analyses are important in showing interactions between actors in healthcare systems such as clinicians and actors in innovative biomedical science, thus drawing attention to the crucial issue of the mechanisms of translation from innovation process to health system. The ecological metaphor draws attention to the 'spaces' and boundaries of biomedical science - their complexities, pathways, and disruptive influences - allowing exploration of how global innovation works on different levels at particular sites, be they laboratories, regions or transnational networks [75,76]. Such a perspective on innovation processes can thus provide for an ecological approach to the interpenetrating levels, links and scales of analysis required to understand the practices of globalised *innovation *processes, and thus the multi-level and multidimensional *governance *issues that they present and with which they interact, which were described in the political economy sections of this discussion.

It is thus necessary to build on concepts like these that enable concepts of innovation and of governance to be linked together. In STS, a number of concepts that indicate recursiveness or 'mutual constitution' have come to the fore. In particular, the concept of *co-production *is now widely used. This broadbrush concept can be seen as underlying much of the discussion in the previous sections. It is of course used in a variety of different ways and embraces a variety of different co-producing entities, though typically these are 'technology' or 'science', and 'society'. Jasanoff [26] in a foundational statement of the co-production 'idiom', identifies two phenomena in particular that we take to be of particular value in addressing biomedical innovation and governance under globalisation: first, the 'emergence and stabilisation' of objects and practices, such as scientific knowledge and technological products; second, 'intelligibility and portability', which we can take to refer to communicability and standardisation, and methods and tools that can provide credibility as 'objects and practices' circulate between different sites and cultures (and, we can add - nation states). Here, we can note the applicability of such concepts to the analysis of transnational governance presented above, for example in the case of TRIPS, where common methods of commoditising and valuing knowledge products have been formed through political negotiation.

Closely related to the concepts of emergence and stabilisation of new sectors, and the boundary-crossing standardisation of knowledge and technologies, are a family of STS concepts with a focus more on how technologies develop and change, focused noticeably around notions that can be summarised as momentum and critical mass. These include concepts such as: technology transitions, path dependence, lock-in, and 'emerging irreversibilities'. The basic principle here is that innovative biomedical technologies may follow distinctive innovation pathways as they emerge, and thus that they may be amenable to different forms of governance intervention and steerage. Technologies may pass a point of no return - 'irreversibility' [77] due to path dependence and lock-in [78], when new practices become built-in to practitioner communities. Thus here again, we can identify a concept that is applied in a technology-specific way which is complementary to the more generic multi-level, multi dimensional political economy described in previous sections. Such complex governance strategy must take account of more 'grass-roots' or niche-level individual science or technology dynamics.

Innovation pathways have also been shown to be shaped by actors' expectations, visions and imagined futures [79]. Expectations 'drive technical and scientific activity, warranting the production of measurements, calculations, material tests, pilot projects and models' [80]. Expectations can be intrinsic to the standardisation processes highlighted in the idiom of co-production, for example, formulating an expectation about the usefulness of a tool or a procedure, can amount to an implicit guide to others to adopt it - an example of portability in action. Expectations may have their own trajectories [81]. As Brown notes, expectations may be at their most powerfully path-shaping in the early stages of technological development, though they may also be subject to de-stabilisation, for example through the promotion of counter-visions or resistance from potential users, consumers, patients or citizens. Thus states, whether they be developmental or competition states, which are grappling with biomedical innovation policy, have the opportunity to consider what forms of strategic intervention they might make in supporting particular visions of bioscientific and technology development as part of 'techno-national' projects, and may shape strategies in terms of the particular timing of intervention in the crucial, path-defining early stages of scientific-technological innovation processes.

A key feature of future-oriented visions that is key to the development of a new biomedical technology or product is the clinical usership [82] constituted in the health system. Various studies have shown the crucial importance of the link between producers and users in shaping new technological developments, for example in diagnostic and imaging technology [83]. This has been formulated as a general insight important to the shaping of innovation trajectories: 'processes bridging the medical supply industry and the communities of medical practitioners' [84]. 'Configuring the users' - see [85] for example - is thus a key part of the active shaping of emerging technologies, zones, sectors and markets, hence the importance of the dynamic between users and producers (user-producer interaction and upstream engagement) in shaping the evolving innovation pathway. State institutions also may have a role in this configuring work, as has been demonstrated in the case of inter-national state vaccine policies [86,87].

In summary, this section has shown that there are a range of valuable concepts produced in innovation studies and STS that should be set alongside a political economy analysis of state strategies in the context of global multilevel, multidimensional governance. Many of these concepts are especially useful in pointing to the individual character of emerging fields or technologies, of which state strategies must take account. Our discussion now turns to consider a second strand of conceptual analysis from STS, namely the idea of co-production, which we develop in terms of understanding the emergence of new - biomedical - fields.

## Co-production of innovation-governance: technological zones and governation

The previous section has shown that a key concept in approaching science and technology innovation under globalisation is that of ecology. It also introduced the crucial topic of emerging, sectoral, standardisable and portable innovations and the importance of actors' expectations and visions of usership as sociotechnical domains where states can, and do, intervene. In this section, the processes of *emergence *of new fields such as those of biomedicine, and the possibilities of their governance, are focused on in more detail. In globalisation theory, emerging forms of national state-global governance have been characterised as 'multi-scalar' [88], and the notion of 'nodal governance' [89] points in the same direction. These formulations too are essentially ecological concepts, thus the concept of ecology can be extended to apply also to governance, that is, the extended, multi-actor, multi-node, 'modular', network governance that is emerging globally, though, as the foregoing analysis of the Rising Powers and developmental states has shown, *unevenly *in different states and biomedical sectors. This approach highlights structural concepts of levels of governance activity, the identification of the more or less heterogeneous range of salient actor networks for given knowledge/technology domains, and aspects of the size and intensiveness of governance actors and activity. In other words, such overarching ecological notions allow a conceptualisation encompassing elements both of 'level' of governance activity (e.g. micro practice/meso institution/macro policy - [90]) and the geographically distributed interaction of multiple innovation-governance actors in the global political economy. In the case of biomedicine such geographies include, for example bioregions (e.g. Singapore) and transnational actors, as well as the nation state dynamics that we have illustrated.

A key element in such strategies is the need for an awareness of the emergence of new 'sectors' and their steerage by nation states in interaction with transnational entities in the context of the global innovation landscape. In this context, a constructionist social theory perspective supports the insight that regulatory governance is one key driver that contributes to the defining of the boundaries of scientific and technological jurisdictions which can be supported, funded, structured, organised, standardised, contested and governed by the state. This insight is somewhat neglected in innovation studies [91]. An under-specification of the innovative, constructive aspects of regulatory work can be seen, for example, in the theorisation of sectoral systems of innovation and production referred to above [63]. In the case of the European Union polity, for example, it has been argued that 'the EU's governance blend... requires... domains to be constituted in order that they may be governed' [[92]: 146]. Novel, hybrid and combinatorial technologies present policy with the need to alter the boundaries between existing institutional arrangements and devise new administrative units. Economic and political interests are of course integral to the formation of such domains and are likely to be the object of conflict between sectoral and national state interests, as has been the case with controversy over TRIPS for example, as noted above. A study of such multi-level conflictual processes of negotiation of regulatory and innovation policy can afford insights into the formation of the rules of engagement for emerging technology zones or sectors. Jurisdictional boundaries, such as define the scope of a technology (e.g. 'tissue engineering') or a governance domain (e.g. 'enterprise' or 'public health') can be difficult to establish in political processes, reflecting conflicting framings of economically important emerging zones.

The jurisdictional fields of technology, knowledge and productiveness that regulation attempts to define can usefully be conceptualised as 'zones' (following Barry [93,94]). The fluid patchwork of regulation interacts with the negotiation of technological zones, driven by various interests and actors. For example, Europe as a trade area itself is partly constituted by regulatory and standard-setting activity: '...technological zones are the objects of developing forms of transnational regulation' [93]. Such zones themselves are partly the product of the work of regulatory policymaking and the active application of regulatory standards. Policy for technical standardisation is a *sine qua non *of the formation of technological zones [95]. The example of the UK's Stem Cell Bank noted above is a good example of such an initiative. By highlighting the embryonic status of emerging technological fields and nascent industries, the concept of technological zone is able to focus on this upstream, stakeholder and state-orchestrated regime-building work that shapes the rules of engagement of an emergent field [82]. At this point, we can note a strong convergence with the concept of a 'horizontal network' of informal rules which flow across national jurisdictions, discussed in our section above on transnational governance [38]. Recognised sectors with established institutional and politico-economic status, such as trade associations, often themselves act as powerful stakeholders in negotiations that construct and shape the pathway of the newly emerging technological zones, and which must find some form of articulation simultaneously with a multitude of national state manoeuvrings [82].

The definition of a technological zone has some flexibility - for example, it may or may not be commensurate with a political territory, be it a nation state or other form or level of jurisdiction. Zones, it has been argued, make association between participants possible but also create new distinctions and separations. In Barry's terms they are 'spaces of circulation in which technologies take more or less standardised forms' [[93]: 122], and in which intellectual property implies new 'objects of technical practice'. Political actors such as states have a role in drawing and legitimating these boundaries and entry criteria. The definition of the 'scope' of an emerging technology or technological product, as can be accomplished through new legislation, can be an integral action in the standardisation and stabilisation of a new zone such as those appearing in biomedicine, such as medical nanotechnology. Key actors, including state institutions, seek to stabilise emerging fields through processes such as the support, mandating and orchestration of rules of engagement, credentialing, incentivising and market-defining. Arguably, zones can thus be seen as precursors to sectors or part of '*sectorisation*' processes in which governance actors, amongst others, are involved (for example, in what sense is there a 'regenerative medicine industry' or a 'tissue engineering industry' that competitive state institutions and industry actors can orient themselves to?). Distributed innovation systems in the era of open innovation do not correspond with traditional national or even sectoral boundaries. There is competition and collaboration at a global level in which national and regional strategies are paramount, as seen in the foregoing sections. In seeking preconditions of newly emergent sectors, the conceptual and empirical project here dovetails with research problems identified from the evolutionary economics/science & technology policy research tradition on 'technological paradigms': 'Finding paradigms after they have become established seems to be reasonably easy. But how to catch them as they form, and manage the formation and establishment of new ones, remain very poorly understood and under-researched.' [96]. The concept of technological zone thus responds to the ecological metaphor and offers a number of advantages, in certain cases, over kindred notions such as industrial sector and technoscientific innovation network (discussed elsewhere: [82]), in approaching this 'how to catch them as they form' problem.

There has been a tendency in innovation/governance studies to assume that innovation precedes regulation, but we take it that this dynamic varies by scientific-technological zone or sector, as suggested by the STS analyses described here, requiring elaboration through empirical case study. Thus there are examples of emerging zones where regulation has preceded the appearance of products (e.g. gene therapy) and where the early product emergence and development of regulation have been more or less simultaneous (e.g. tissue engineered products). The open-ended nature of scientific and technological development means that matters such as the legal definition of a technology within a given jurisdiction are often left open-ended in newly formulated legislation.

In order to articulate the broad view illustrated here that governance is a constructive process shaping the emergence of new bio-objects, and in part being shaped by technology innovation and potential markets (as well as being an external, steering and controlling force) we have proposed elsewhere the concept of 'governation' (*govern*ance + innov*ation*, see[82]). Like 'co-production', the term hides more than it reveals, its main purpose being to highlight a range of mutually-constituting forces which require detailed case study, such as may be provided by the emerging strands of biomedical science and technology in the global, and particularly the Rising Powers context. Many of these substantive forces that constitute the co-producing, multiple dimensions of science, society and the market in the biomedical governance field have been identified, by way of illustration, in the foregoing sections of this discussion.

## Conclusions: the conceptual challenge for global biomedical innovation-governance

To recap the political economy perspective, it is a state's ability to respond to global opportunities and the frequently transnational nature of technological innovation, rather than the coherence of its inward looking policies, that provides the key to its likely position in the biomedical future and its chances of improving its global geopolitical position. Contemporary knowledge economies are invariably global and this is reflected in the transnational movement of the scientific and financial capital that fuel them. More so than ever, no state can afford to be a political island. Increasingly it is the production of policies that facilitate an advantageous engagement with global forces that will determine a state's effectiveness in innovative fields such as biomedicine, not the pursuit of protective policies that are bound to be undermined by the growing presence of international economic governance. Successful geopolitical manoeuvring by states is more likely to be characterised by the constructive use of permeable borders than the rigid application of sovereign jurisdiction.

Indeed, it is one of the signal lessons of globalisation that the permeability of state boundaries can be turned to a state's geopolitical advantage. In their review of the opportunities for biotech companies in two Rising Powers countries, India and China, Goodall and colleagues remark on the possibility that China and India are opening up a new model of biotech development: 'Call it the "modular model", a kind of decentralised R & D system where different aspects of R & D are distributed globally and conducted almost autonomously in different locations' [97]. Because their innovation needs are different from those of the competition states of the West, China and India will inevitably push the dynamic of globalisation in directions that suit their interests and their particular strategies on the knowledge production process. In the case of biomedical innovation, there are already strong indications that both countries will play to their knowledge production strengths in terms of the availability of research materials (e.g. oocytes - [98]), a large and diverse pool of human subjects for clinical experimentation (clinical labour), and inexpensive scientific labour. In return they will want to obtain such advantages as access to the basic science in the field, a division of the patenting benefits, and a sustained supply of venture capital. At the same time we can expect that their strategies on the emerging governance of biomedical innovation through such mechanisms as TRIPS and international ethical guidelines will become considerably more proactive.

Concepts of the inter-linked, co-producing ecology of innovation and governance processes emphasise the permeability of state boundaries and the multidimensional, multi-sited, multi-scalar nature of innovation-governance in global biomedicine. This paper has provided an overview of a range of approaches to understanding issues of innovation and governance, which can be applied to the case of global biomedical innovation. We have pointed out how particular insights from this range of approaches are relevant to global biomedical innovation, and suggested some of the lines of convergence and complementarity between the somewhat disparate social science approaches. The challenge now is to further develop approaches to the issue of the Rising Powers' entry into the globalising biomedical political economy that take these convergences further, to combine analysis of politico-economic states' competitive interests and strategies with an analysis of the dynamics of, and the steerage or 'modulation' [99] of, emerging international scientific-technological zones and sectors, taking account of both public governmental and private regulatory possibilities.

To summarise and bring together the threads of our concept-mapping project, we return to our 'triangle of tensions' as a heuristic to describe the main broad dimensions of biomedical governance. Combining the triangle of governance arenas (science, society and the market), levels of governance (transnational, national and sub-national) and modes of governance (public and private) produces a *multi-dimensional *framework for the analysis of biomedical innovation (Table 1). We do not attempt here to fill in each cell, though some examples can be given. Thus, a transnational private governance actor in the 'science' dimension is the International Conference on Harmonisation (ICH, the global pharmaceutical regulatory body); national-level public governance actors in the 'society' dimension are, for example, the UK's Human Fertilisation and Embryology Authority (HFEA), or the National Bioethics Committee of India.

**Table 1:** Multi-dimensional co-production of governance and innovation

	*Level of governance*					
	***Transnational***		***National***		***Sub-national***	

***Innovation-Governance arenas***	*Public*	*Private*	*Public*	*Private*	*Public*	*Private*

*Science*						

*Society*						

*Market*						

This multi-dimensional framework can move towards enabling a description of the task of governing a set of interlinking national and transnational markets that together constitute the dynamic of knowledge production: the funding market of scientific research, the scientific labour market, the moral economy of ethics for the trading of regulatory values [100], the intellectual property market and the financial venture capital market - all energised also by the political competition. Change in one market is likely to impact on another thus generating a need for a self-sustaining system of multi-dimensional governance that has both a stable core capacity at transnational and state levels and the ability to flex and evolve its own mechanisms, particularly through the invention of networked private governance.

This paper suggests that the future further development of the framework should meet a number of criteria that include:

1. An analysis of how governance of particular sectors, zones, scientific fields or technologies (in other words, the issues discussed in the last two sections of this paper, on Ecologies and Co-production) articulate with the multidimensional framework of the political economy of transnational and national innovation-governance profiles, and application of this analysis to particular emerging subsectors of biomedicine, such as stem cell research, tissue engineering, molecular diagnostics and stratified/personalised medicine;

2. Recognition of the current global balance of power in biomedical innovation characterised by a US hegemony and an emerging challenge from the Rising Powers;

3. Incorporation of data on the emerging innovation activity of the Rising Powers actors (e.g. the mobilisation of human expertise and material resources), and of the dynamic relationships between the Rising Powers and other global innovation and governance actors, both state and non-state;

4. The contribution of the historical trajectory of private and public innovation and governance institutions, in the national, sectoral and technological zone context;

5. The significance of sociocultural and ethical issues and actors impinging on and shaping innovation and governance processes, including civil society groups and the representation of ethical interests, and acting at different governance levels;

6. Issues of how 'demand' for knowledge and products, and the construction of 'need' and clinical usership in health systems and health economies are addressed by states with varying strategies for accessing value chains through known markets or new market-building;

7. The impact of path-shaping expectations and institution formation between biomedicine producers (firms, academia) and users (healthcare systems, patients) and governments' strategic involvement in them, involving both competitive and collaborative dimensions;

8. And the need to identify points and arenas of potential intervention, negotiation and conflict between the Rising Powers and other states in the different levels and nodes of the transnational governance of biomedical innovation, both public and private.

## Competing interests

The authors declare that they have no competing interests.

## Authors' contributions

The paper was conceived jointly by BS and AF. BS primarily wrote sections 1-4, AF primarily wrote sections 5-6. BS and AF co-wrote the Introduction and Conclusion. AF revised the paper. All authors read and approved the final manuscript.

## References

[B1] Hogarth S Salter B Regenerative medicine in Europe: global competition and innovation governance Regenerative Medicine 2010 5 6 971 85 10.2217/rme.10.81 21082895

[B2] Cerny P Paradoxes of the competition state: the dynamics of political globalisation Government and Opposition 1997 32 2 251 74 10.1111/j.1477-7053.1997.tb00161.x

[B3] Hay C Re-stating politics, re-politicising the state: neo-liberalism, economic imperatives and the rise of the competition state Political Quarterly 2004 75 1 38 50 10.1111/j.1467-923X.2004.621_1.x

[B4] Hirsch J Jessop B From the Fordist to the post-Fordist state The politics of flexibility: restructuring state and industry in Britain, Germany and Scandinavia 1991 Edward Elgar, Aldershot

[B5] Jessop B The future of the capitalist state 2002 Polity Press, Oxford

[B6] Messner D The network society: economic development and international competitiveness as problems of social governance 1997 Frank Cass, London

[B7] Pierre J (ed) Debating governance 2000 Oxford University Press, Oxford

[B8] Asheim BT Gertler MS Fagerberg J, Mowery DC, Nelson RR The geography of innovation: regional innovation systems The Oxford handbook of innovation 2004 Oxford University Press, Oxford

[B9] Cooke P The evolution of biotechnology in three continents: Schumpeterian or Penrosian? European Planning Studies 2003 11 7 757 63 (Editorial) 10.1080/0965431032000121328

[B10] Cooke P Regional knowledge capabilities, embeddedness of firms and industry organisation: bioscience megacentres and economic geography European Planning Studies 2004 11 7 625 41 10.1080/0965431042000219987

[B11] Hart D Private technological capabilities as products of national innovation systems: four ways of looking at the state Science and Public Policy 2002 29 3 181 88 10.3152/147154302781780985

[B12] Hansen A Biotechnology regulation: limiting or contributing to biotech development? New Genetics and Society 2001 20 3 255 71 10.1080/14636770120093010

[B13] Laredo P Mustar P Laredo P and Mustar P General conclusion: three major trends in research and innovation policies Research and innovation policies in the global economy: an international comparative perspective 2001 Edward Elgar, Cheltenham

[B14] Applebaum RP Henderson J (eds) States and development in the Asian Pacific Rim 1992 Newbury Park CA, Sage

[B15] Hawes G Liu H Explaining the dynamics of the southeast Asian political economy: state, society and the search for economic growth World Politics 1993 45 4 629 60 10.2307/2950711

[B16] Onis Z The logic of the developmental state Comparative Politics 1991 24 1 109 26 10.2307/422204

[B17] Johnson C MITI and the Japanese miracle 1982 Stanford University Press, Stanford

[B18] Wade R What strategies are viable for developing countries today? The World Trade Organisation and the shrinking of 'development space Review of International Political Economy 2003 10 4 621 44 10.1080/09692290310001601902

[B19] Weiss L Developmental states in transition: adapting, dismantling, innovating, not 'normalising' The Pacific Review 2000 13 1 21 55 29 10.1080/095127400363631

[B20] Mann M Has globalisation ended the rise and rise of the nation state? Review of International Political Economy 1997 4 3 472 96 10.1080/096922997347715

[B21] Weiss L The myth of the powerless state 1998 Cornell University Press, Ithaca

[B22] Kim YT Neoliberalism and the decline of the developmental state Journal of Contemporary Asia 1999 29 4 441 62 10.1080/00472339980000231

[B23] Wu Y Rethinking the Taiwanese developmental state The China Quarterly 2004 177 91 114 10.1017/S0305741004000062

[B24] Wong J Re-making the developmental state in Taiwan: the challenges of biotechnology International Political Science Review 2005 26 169 191 10.1177/0192512105050380

[B25] Salter B Salter C Governing innovation in the biomedicine knowledge economy: stem cell science in the USA Science and Public Policy 2010 37 2 87 100 10.3152/030234210X489617

[B26] Jasanoff S States of knowledge: co-production of science and social order 2004 Routledge, London

[B27] Hooghe L Marks G Unravelling the central state, but how? Types of multi-level governance American Political Science Review 2003 97 233 43

[B28] Büthe T Governance through private authority? Non-state actors in world politics Journal of International Affairs 2004 58 281 90

[B29] Knill C Lehmkuhl D Private actors and the state: internationalisation and changing patterns of governance Governance 2002 15 1 41 63 10.1111/1468-0491.00179

[B30] Hodge G Coghill K Accountability in the privatized state Governance 2007 20 4 675 702 10.1111/j.1468-0491.2007.00377.x

[B31] Scott C Private regulation of the public sector: a neglected facet of contemporary governance Journal of Law and Society 2002 29 1 56 76 10.1111/1467-6478.00211

[B32] Tait J Wield D Bruce A Chataway J Health biotechnology to 2030. OECD International Futures Project on The bioeconomy to 2030: designing a policy agenda 2007 OECD, Paris

[B33] Coleman WD Perl E Internationalised policy environments and policy network analysis Political Studies 1999 47 691 709 10.1111/1467-9248.00225

[B34] Rosenau JN Czempiel E Governance without government: order and change in world politics 1992 Cambridge University Press, Cambridge

[B35] Young OR Governance in world affairs 1999 Cornell University Press, Ithaca NT

[B36] Etzioni A Beyond transnational governance International Journal 2001 Autumn 595 610 10.2307/40203606

[B37] Ronit K Schneider V Global governance through private organisations Governance 1999 12 3 243 66 10.1111/0952-1895.00102

[B38] Rosenau JN J Pierre Change, complexity and governance in globalising space Understanding governance 2000 Oxford University Press, Oxford 168 200

[B39] Rosenau J Distant Proximities: Dynamics Beyond Globalization 2003 Princeton University Press, Princeton

[B40] Isasi RM Knoppers BM Governing stem cell banks and registries: Emerging Issues Stem Cell Research 2009 3 2-3 96 105 10.1016/j.scr.2009.05.003 19586808

[B41] Shorrett P Rabinow P Billings PR The changing norms of the life sciences Nature Biotechnology 2003 21 123 25 10.1038/nbt0203-123 12560827

[B42] Mowery D Sampat B The Bayh-Dole Act of 1980 and university-industry technology transfer: a model for other OECD governments? The Journal of Technology Transfer 2004 30 1-2 115 27 10.1007/s10961-004-4361-z

[B43] Rai A Eisenberg R Bayh-Dole reform and the progress of biomedicine Law and Contemporary Problems 2003 66 289 314

[B44] Forero-Pineda C The impact of stronger intellectual property rights on science and technology in developing countries Research Policy 2006 35 808 24 10.1016/j.respol.2006.04.003

[B45] Salter B Governing stem cell science in China and India: emerging economies and the global politics of innovation New Genetics and Society 2008 27 2 145 49 10.1080/14636770802077090

[B46] Salter B State strategies and the global knowledge economy: the geopolitics of regenerative medicine Geopolitics 2009 14 1 31 10.1080/14650040802578666

[B47] Beeson M American hegemony and regionalism: the rise of East Asia and the end of the Asia-Pacific Geopolitics 2006 11 4 541 60 10.1080/14650040600890727

[B48] Haque MS Governance and bureaucracy in Singapore: contemporary reforms and implications International Political Science Review 2004 25 2 227 10.1177/0192512104042314

[B49] Wong J The adaptive developmental state in East Asia Journal of East Asian Studies 2004 4 345 62

[B50] Lee CS Schrank A Incubating Innovation or Cultivating Corruption?: The Developmental State and the Life Sciences in Asia Social Forces 2010 88 3 1231 1255 10.1353/sof.0.0282

[B51] Minns J Of miracles and models: the rise and decline of the developmental state in South Korea Third World Quarterly 2001 22 6 1025 43 10.1080/01436590120099777

[B52] Saich T The changing role of government? 2004 Background Note for the World Bank Report on China's 11th Five Year Plan http://ksghome.harvard.edu/~asaich/The_Changing_Role_of_Government.pdf

[B53] Baum R Shevchenko A Goldman M, MacFarquar R The state of the state The paradox of reform of China's post-Mao reforms 1999 Harvard University Press, Cambridge MA

[B54] Saich T Benewick R et al Reform and the role of the state in China Asian politics in development 2003 Frank Cass, London

[B55] Dahlman C Aubert J China and the knowledge economy: seizing the 21st century 2001 Washington; World Bank http://info.worldbank.org/etools/docs/library/137742/ChinaKE.pdf

[B56] Chibber V Locked in place: state building and late industrialisation in India 2003 Princeton University Press, Princeton

[B57] World Bank India: Five Years of Stabilization and Reform and the Challenges Ahead 1997 New York: World Bank

[B58] Dahlman C Utz A India and the knowledge economy: leveraging strengths and opportunities 2006 Washington: World Bank http://info.worldbank.org/etools/docs/library/145261/India_KE_Overview.pdf

[B59] Winters A Yusuf S Dancing with giants: China, India and the global economy 2007 Institute of Policy Studies, Singapore

[B60] Salter B China, globalisation and health biotechnology innovation: venture capital and the adaptive state East Asian Science and Technology: an International Journal 2009 3 4 401 425 10.1007/s12280-009-9090-9

[B61] Freeman C The National System of Innovation in Historical Perspective Cambridge Journal of Economics 1995 19 5 24

[B62] Bartholemew S National systems of biotechnology innovation: complex interdependence in the global system Journal of international Business Studies 1997 28 2 241 66 10.1057/palgrave.jibs.8490100

[B63] Malerba F New Challenges for Sectoral Systems of Innovation in Europe Presented at DRUID Conference on National Innovation Systems, Industrial Dynamics and Innovation Policy 1999 Rebild

[B64] Malerba F Innovation in sectoral systems: What we know... and what we would like to know Presentation to SPRU 40th Anniversary Conference 2006 University of Sussex, UK

[B65] Carlsson B Stankiewicz R On the nature, function and composition of technological systems Journal of Evolutionary Economics 1991 1 93 118 10.1007/BF01224915

[B66] Nelson RR Winter SG In search of a useful theory of innovation Research Policy 1977 6 1 36 76 10.1016/0048-7333(77)90029-4

[B67] Nelson RR Winter SG An Evolutionary Theory of Economic Change 1982 Harvard University Press, Cambridge MA

[B68] Pavitt K Sectoral patterns of technical change: towards a taxonomy and a theory Research Policy 1984 13 343 373 10.1016/0048-7333(84)90018-0

[B69] Dosi G Technological paradigms and technological trajectories. A suggested interpretation of the determinants and directions of technical change Research Policy 1982 11 3 147 162 10.1016/0048-7333(82)90016-6

[B70] Leigh Star S Ecologies of knowledge: work and politics in science and technology 1995 State University of New York Press, Albany

[B71] Greenhalgh T Robert G Macfarlane F Bate P Kyriakidou O Diffusion of innovations in service organizations: Systematic review and recommendations Milbank Quarterly 2004 82 581 629 10.1111/j.0887-378X.2004.00325.x 15595944 PMC2690184

[B72] Metcalfe JS James A Mina A Emergent innovation systems and the delivery of clinical services: The case of intra-ocular lenses Research Policy 2005 34 9 1283 1304 10.1016/j.respol.2005.01.015

[B73] Ramlogan R Mina A Tampubolon G Metcalfe JS Networks of knowledge: The distributed nature of medical innovation Scientometrics 2007 70 2 459 89 10.1007/s11192-007-0212-7

[B74] Ramlogan R Consoli D Knowledge, understanding and the dynamics of medical innovation. Munich Personal RePEc Archive 2008 http://mpra.ub.uni-muenchen.de/9112/1/MPRA_paper_9112.pdf

[B75] Livingstone DN Putting science in its place: geographies of scientific knowledge 2003 University of Chicago Press

[B76] Wainwright SP Williams C Spaces of speech and places of performance: an outline of a geography of science approach to embryonic stem cell research and diabetes New Genetics and Society 2008 27 161 173 10.1080/14636770802077116

[B77] van Merkerk RO van Lente H Tracing emerging irreversibilities in emerging technologies: The case of nanotubes Technological Forecasting and Social Change 2005 72 9 1094 111 10.1016/j.techfore.2004.10.003

[B78] Rycroft RW Kash DE Path Dependence in the Innovation of Complex Technologies Technology Analysis & Strategic Management 2002 14 1 21 35 10.1080/09537320220125865

[B79] Brown N Michael M A Sociology of Expectations: Retrospecting Prospects and Prospecting Retrospects Technology Analysis and Strategic Management 2003 15 1 3 18 10.1080/0953732032000046024

[B80] Borup M Brown N Konrad K van Lente H The Sociology of Expectations in Science and Technology Technology Analysis & Strategic Management 2006 18 3/4 285 298 10.1080/09537320600777002

[B81] Geels F Raven R Non-linearity and Expectations in Niche-Development Trajectories: Ups and Downs in Dutch Biogas Development (1973-2003) Technology Analysis & Strategic Management 2006 18 3/4 375 392 10.1080/09537320600777143

[B82] Faulkner A Medical Technology into Healthcare and Society: a sociology of devices, innovation, and governance 2009 Palgrave Macmillan, Basingstoke

[B83] Blume S Insight and Industry: on the dynamics of technological change in medicine 1992 MIT Press

[B84] Metcalfe S James A McMeekin A Distributed Innovation Processes and the Uneven Growth Of Medical Knowledge IHT Programme Research Findings 2004 http://www.york.ac.uk/res/iht/researchfindings/MetcalfeFindings.pdf

[B85] Oudshoorn N Pinch TJ How users matter: the co-construction of users and technologies 2003 MIT Press, Cambridge/London

[B86] Blume S Lock in, the state and vaccine development: Lessons from the history of the polio vaccines Research Policy 2005 34 159 173 10.1016/j.respol.2004.12.001

[B87] Blume S Zanders M Vaccine independence, local competences and globalisation: Lessons from the history of pertussis vaccines Social Science & Medicine 2006 63 1825 1835 10.1016/j.socscimed.2006.04.014 16764977

[B88] Sassen S Participation of states and citizens in global governance Indiana Journal of Global Legal Studies 2003 10 1 5 28 10.2979/GLS.2003.10.1.5

[B89] Burris S Drahos B Shearing C Nodal Governance Australian Journal of Legal Philosophy 2005 30 58

[B90] Swan J Newell S Robertson M Goussevskaia A Bresnen M The role of institutional differences in biomedical innovation processes: a comparison of the UK and US International Journal of Healthcare Technology and Management 2007 8 3-4 333 53 10.1504/IJHTM.2007.013167

[B91] Faulkner A Regulatory policy as innovation: constructing rules of engagement of a technological zone for tissue engineering in the European Union Research Policy 2009 38 4 637 646 10.1016/j.respol.2009.01.017

[B92] Delanty G Rumford C Rethinking Europe: social theory and the implications of Europeanization 2005 Routledge, London

[B93] Barry A Political Machines: Governing a Technological Society 2001 The Athlone Press, London

[B94] Barry A Technological Zones European Journal of Social Theory 2006 9 2 239 253 10.1177/1368431006063343

[B95] Callon M Europe wrestling with technology Economy and Society 2004 33 1 121 134 (Review of Barry A: Political Machines, 2001) 10.1080/0308514042000176766

[B96] von Tunzelmann N Malerba F Nightingale P Metcalfe S Technological paradigms: past, present and future Industrial and Corporate Change 2008 17 3 467 484 10.1093/icc/dtn012

[B97] Goodall S Janssens B Wagner K Wong J Woods W Yeh M The promise of the East: India and China as R & D options Nature Biotecnology 2006 24 9 1064 10.1038/bioent910 17061321

[B98] Gottweis H Salter B Waldby C The global politics of human embryonic stem cell science: regenerative medicine in transition 2009 Palgrave Macmillan, Basingstoke

[B99] Rip A Co-evolution of science, technology and society 2002 http://www.sciencepolicystudies.de/dok/expertise-rip.pdf Accessed April 2010

[B100] Salter B Salter C Bioethics and the global moral economy: the cultural politics of human embryonic stem cell science Science, Technology and Human Values 2007 32 5 1 28 10.1177/0895904805303202

